# Interhemispheric Difference Images from Postoperative Diffusion Tensor Imaging of Gliomas

**DOI:** 10.7759/cureus.817

**Published:** 2016-10-05

**Authors:** Robert Kosztyla, Stefan A Reinsberg, Vitali Moiseenko, Brian Toyota, Alan Nichol

**Affiliations:** 1 Department of Physics and Astronomy, University of British Columbia; 2 Department of Medical Physics, BC Cancer Agency; 3 Department of Radiation Medicine and Applied Sciences, University of California, San Diego; 4 Division of Neurosurgery, University of British Columbia; 5 Vancouver Centre, BC Cancer Agency

**Keywords:** glioma, diffusion tensor imaging, magnetic resonance imaging, image analysis, target volumes

## Abstract

Introduction

Determining the full extent of gliomas during radiotherapy planning can be challenging with conventional T1 and T2 magnetic resonance imaging (MRI). The purpose of this study was to develop a method to automatically calculate differences in the fractional anisotropy (FA) and mean diffusivity (MD) values in target volumes obtained with diffusion tensor imaging (DTI) by comparing with values from anatomically homologous voxels on the contralateral side of the brain.

Methods

Seven patients with a histologically confirmed glioma underwent postoperative radiotherapy planning with 1.5 T MRI and computed tomography. DTI was acquired using echo planar imaging for 20 noncolinear directions with b = 1000 s/mm^2^ and one additional image with b = 0, repeated four times for signal averaging. The distribution of FA and MD was calculated in the gross tumor volume (GTV), shells 0-5 mm, 5-10 mm, 10-15 mm, 15-20 mm, and 20-25 mm outside the GTV, and the GTV mirrored in the left-right direction (mirGTV). All images were aligned to a template image, and FA and MD interhemispheric difference images were calculated. The difference in mean FA and MD between the regions of interest was statistically tested using two-sided paired t-tests with α = 0.05.

Results

The mean FA in mirGTV was 0.20 ± 0.04, which was larger than the FA in the GTV (0.12 ± 0.03) and shells 0-5 mm (0.15 ± 0.03) and 5-10 mm (0.17 ± 0.03) outside the GTV. The mean MD (×10^-3^ mm^2^/s) in mirGTV was 0.93 ± 0.09, which was smaller than the MD in the GTV (1.48 ± 0.19) and the peritumoral shells. The distribution of FA and MD interhemispheric differences followed the same trends as FA and MD values.

Conclusions

This study successfully implemented a method for calculation of FA and MD differences by comparison of voxel values with anatomically homologous voxels on the contralateral side of the brain. Further research is warranted to determine if radiotherapy planning using these images can be used to improve target delineation.

## Introduction

In the radiotherapy planning of high-grade gliomas, the traditional gross tumor volume (GTV) is the gadolinium contrast enhancement on magnetic resonance imaging (MRI) [1]. Enhancement of high-grade gliomas on T1-weighted images arises from blood-brain barrier disruptions that give abnormal vessel permeability to gadolinium [1-2]. However, pathological studies have identified glioma cells infiltrating the brain beyond the area of contrast enhancement [3]. Studies have shown that the extent of glioma cell infiltration, which is not visible on conventional MRI with gadolinium contrast enhancement, can be identified using diffusion tensor imaging (DTI) [4-5]. In addition, DTI has been used to define anisotropic, patient-specific GTV-to-clinical target volume (CTV) margins in numerical simulations [6-7].

DTI maps and characterizes the three-dimensional distribution of anisotropic diffusion of water in the brain. DTI measures often reported are the mean diffusivity (MD) and the fractional anisotropy (FA). MD reflects the magnitude of diffusion and is often referred to as the apparent diffusion coefficient (ADC). FA is a value that corresponds to the degree of anisotropy of diffusion, where FA = 0 indicates diffusion that is isotropic and FA = 1 indicates the diffusion is restricted to one direction. While FA and MD values have been associated with glioma cell density and proliferation activity [8-11], FA and MD images can be difficult to interpret for postoperative radiation therapy planning.

A potential method to improve interpretation of FA and MD images is by the calculation of interhemispheric difference images. In the method proposed by Aubert-Broche, et al [12], interhemispheric difference images identified regions of functional interhemispheric asymmetries from ^99m^Tc-exametazime and ^99m^Tc-ethyl cysteinate dimer single photon emission computed tomography (SPECT) images of the brain using anatomical information from MRI. Their method matched each MRI voxel to its anatomically homologous voxel on the contralateral side of the brain. By mapping these voxels to the SPECT image, it was possible to compute SPECT interhemispheric difference images.

The application of interhemispheric difference images to DTI would allow the voxel-by-voxel comparison of FA and MD values in a tumor or peritumoral region with their corresponding anatomically homologous voxels on the contralateral side of the brain. By providing information on which voxels differ from expected values obtained from the contralateral size of the brain, this approach may help clinicians to contour disease using FA and MD images during radiotherapy planning. Therefore, the purpose of this study was to implement a method that automatically calculates FA and MD interhemispheric difference images.

## Materials and methods

### Patient selection

Eligible patients were at least 18 years of age, had a contrast-enhancing mass on diagnostic brain CT or MRI that strongly suggested a diagnosis of WHO Grade III or IV glioma prior to surgery, had a Karnofsky Performance Status of 70 or greater, and had a glomerular filtration rate of 60 mL/min or greater. Exclusion criteria were: indication for urgent craniotomy to relieve mass effect, T1 enhancement or T2 signal that involved the basal ganglia, previous intracranial malignancy or any invasive malignancy unless free of disease at least five years, prior cranial irradiation, medication for the treatment of Parkinson’s disease, or allergies or contraindications to contrast MRI or radiation therapy. Our institutional research ethics board approved the study and all subjects provided written informed consent. The National Institutes of Health clinical trial identifier was NCT01248754.

### Imaging

Patients underwent postoperative MRIs acquired using a 1.5 T Siemens Magnetom Symphony Tim system (Siemens Healthcare, Erlangen, Germany). T1-weighted images with gadolinium contrast enhancement using the turbo spin echo sequence (echo time (TE) = 14 ms, pixel resolution = 1 mm, slice thickness = 3 mm) and T2-weighted fluid-attenuated inversion recovery (FLAIR) images were obtained (TE = 97 ms, pixel resolution = 0.5 mm, slice thickness = 3 mm).

DTI was performed using single-shot echo planar imaging for 20 noncolinear directions with *b*= 1000 s/mm^2^ and one additional image with *b*= 0 (TE = 98 ms, repetition time = 3800 ms, 128 × 128 acquisition matrix, pixel resolution = 1.95 mm, slice thickness = 5 mm, slice spacing = 1 mm). The diffusion imaging sequence was repeated four times for signal averaging.

Images were processed using the Oxford Centre for Functional MRI of the Brain Software Library (FSL) (version 5.0; Oxford, UK) [13]. The brain extraction tool was used to obtain a brain mask of the T1-weighted image [14]. DTI data was corrected for eddy current distortions, and then FA and MD images were generated from diffusion tensor fitting [15]. The MD and FA in each voxel were given by:

$$\mathrm{MD}=\frac{\lambda_1+\lambda_2+\lambda_3}{3}$$

$$\mathrm{FA}=\sqrt{\frac{3}{2}\cdot\frac{\sum_{i=1}^3\left(\lambda_i-\textrm{MD}\right)^2}{\sum_{i=1}^3\lambda_i^2}}$$

where λ_1_, λ_2_ and λ_3_ were the eigenvalues of the diffusion tensor.

**Figure 1:**
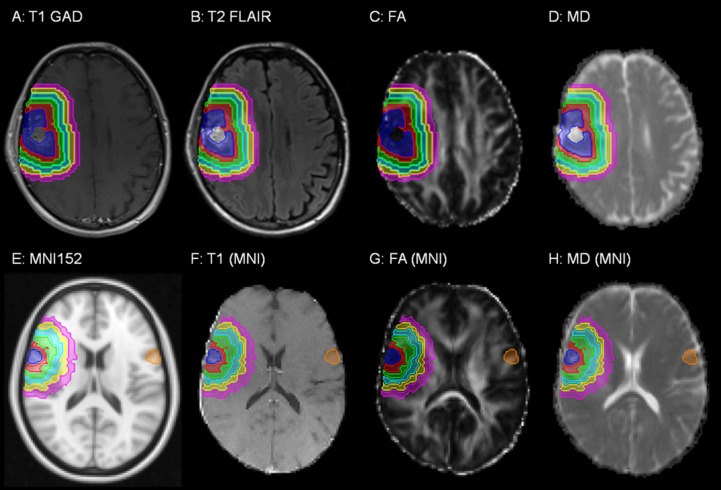
Magnetic resonance images (MRI) obtained for a sample patient. The (A) T1-weighted MRI with gadolinium contrast enhancement, (B) T2-weighted FLAIR MRI, (C) fractional anisotropy (FA), (D) mean diffusivity (MD), (E) the MNI 152 symmetric standard space image, (F) T1-weighted MRI registered to the symmetric standard space image, (G) FA image registered to the symmetric standard space image, and (H) MD image registered to the symmetric standard space image are shown. Regions of interest shown are the GTV (blue), peritumoral shells 0-5 mm (red), 5-10 mm (green), 10-15 mm (cyan), 15-20 mm (yellow), and 20-25 mm (magenta) outside the GTV, and GTV mirrored in the left-right direction (orange).

Each patient's attending radiation oncologist delineated the GTV on T1-weighted and T2-weighted images, excluding the surgical cavity. Peritumoral contours were then defined by three-dimensional isotropic dilation of the GTV by 5 mm, 10 mm, 15 mm, 20 mm, and 25 mm. Peritumoral shells 0-5 mm, 5-10 mm, 10-15 mm, 15-20 mm, and 20-25 mm outside the GTV were then defined using Boolean operations (e.g., the region 0-5 mm outside the GTV was defined by the region inside the GTV + 5 mm contour but outside the GTV). Shells were cropped to ensure that they were within the brain mask automatically generated by the brain extraction tool [14]. All images were registered to the T1-weighted image using a 12-parameter affine model with mutual information cost function [16-17]. Figure 1 shows an example of the registered T1-weighted, T2-weighted, FA, and MD images, as well as the regions of interest for a sample patient.

All images and regions of interest were then aligned to the Montreal Neurological Institute (MNI) 152 symmetric standard space T1-weighted average structure template image (Figure 1E) [18] using the FSL nonlinear registration tool (using an isotropic warp resolution of 10 mm and subsampling factors of 4, 2, and 1). It used a B-spline representation of the registration warp field [19]. The mirror image of the GTV in the left-right direction in the symmetric standard space was defined as "mirGTV". Examples of images aligned to the symmetric standard space image, with mirGTV volumes, are shown in Figure 1.

The distribution of FA and MD values inside the GTV, 0-5 mm, 5-10 mm, 10-15 mm, 15-20 mm, and 20-25 mm shells outside the GTV, and mirGTV were computed using the images aligned in the symmetric standard space with MATLAB, version 7.0.0.19920 (The MathWorks, Inc., Natick, MA). Differences between the mean FA and MD in the mirGTV and the mean FA and MD in the GTV and shells outside the GTV were tested for statistical significance using two-sided paired t-tests (α = 0.05).

### Interhemispheric difference images

**Figure 2:**
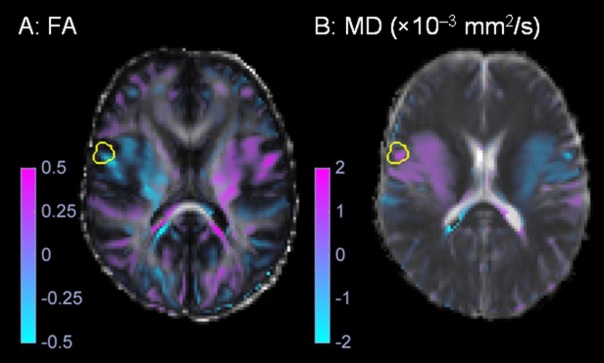
Interhemispheric difference images. An example of the (A) fractional anisotropy (FA) and (B) mean diffusivity (MD) interhemispheric difference images obtained for the sample patient shown in Figure 1. The gross tumor volume is shown in yellow.

Using the FA and MD symmetric standard space images, interhemispheric difference images for FA (ΔFA) and MD (ΔMD) were calculated using the equations:

$$\Delta\mathrm{FA}(x,y,z)=\mathrm{FA}(x,y,z)-\mathrm{FA}(-x,y,z)$$

$$\Delta\mathrm{MD}(x,y,z)=\mathrm{MD}(x,y,z)-\mathrm{MD}(-x,y,z)$$

where FA(x,y,z) and MD(x,y,z) were the FA and MD at voxel (x,y,z) in the symmetric standard space image and FA(-x,y,z) and MD(-x,y,z) were the FA and MD of the corresponding anatomically homologous voxel (-x,y,z) on the contralateral side of the brain. Figure 2 shows a qualitative example of FA and MD interhemispheric difference images. The distributions of FA and MD interhemispheric differences were characterized in the GTV and the shells 0-5 mm, 5-10 mm, 10-15 mm, 15-20 mm, and 20-25 mm outside the GTV. Mean interhemispheric differences were statistically compared using two-sided t-tests (α = 0.05).

## Results

Seven patients enrolled in the study had histologically confirmed, newly diagnosed gliomas and underwent radiotherapy planning with postoperative MRI and computed tomography (CT). Table 1 shows the patient and tumor characteristics, as well as the timing between surgery and postoperative MRI.

**Table 1:** Patient Characteristics

No. of Patients	7
Sex	
Female	2 (29%)
Male	5 (71%)
Age, median (range)	51 (34-80) years
Histology	
Oligoastrocytoma (Grade II)	1 (14%)
Anaplastic astrocytoma (Grade III)	1 (14%)
Glioblastoma (Grade IV)	5 (71%)
Time, median (range)	
Surgery to MRI	6 (2–27) days

**Figure 3:**
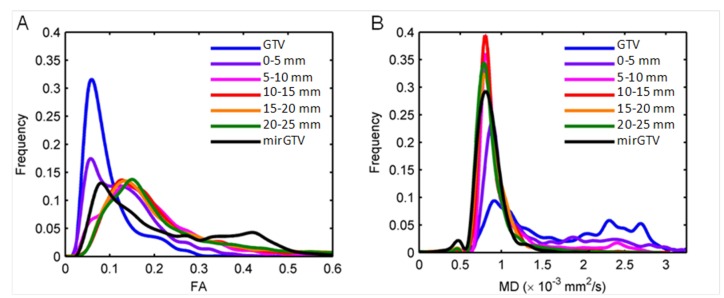
Fractional anisotropy and mean diffusivity distributions. The distribution of (A) fractional anisotropy (FA) and (B) mean diffusivity (MD) values in the gross tumor volume (GTV), peritumoral shells, and mirrored GTV (mirGTV) for a patient with a glioblastoma.

Figure 3 illustrates examples of the distribution of FA and MD in the GTV, 0-5 mm, 5-10 mm, 10-15 mm, 15-20 mm, and 20-25 mm shells outside the GTV and mirGTV. The distribution of FA and MD suggests that FA and MD values approached those of normal brain tissue as the distance outside the GTV increased.

**Figure 4:**
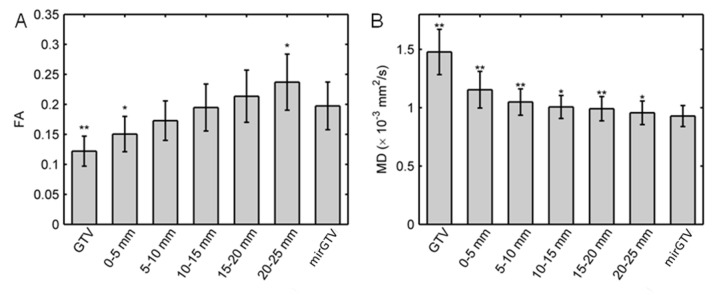
Mean fractional anisotropy and mean diffusivity values. For all patients, the mean (A) fractional anisotropy (FA) and (B) mean diffusivity (MD) values for the gross tumor volume (GTV), peritumoral shells, and mirrored GTV (mirGTV). Error bars show the standard error. Symbols: * *P *< 0.05 and ** *P *< 0.01 for two-sided paired t-tests comparing GTV and peritumoral shells to mirGTV.

Figure 4 shows the mean and standard error of the FA and MD values in each region of interest. The mean FA in the GTV (0.12 ± 0.03) and in the regions 0-5 mm (0.15 ± 0.03) and 5-10 mm (0.17 ± 0.03) outside the GTV were smaller than the mean FA in mirGTV (0.20 ± 0.04). The mean FA in the regions 10-15 mm (0.20 ± 0.04) and 15-20 mm (0.21 ± 0.04) outside the GTV were similar to the mean values in mirGTV. The mean FA in the region 20-25 mm (0.24 ± 0.05) outside the GTV was larger than the value in mirGTV. The mean MD (× 10^-3^ mm^2^/s) was larger in the GTV (1.48 ± 0.19) and regions 0-5 mm region (1.15 ± 0.26), 5-10 mm (1.05 ± 0.11), 0-15 mm (1.01 ± 0.10), 15-20 mm (0.99 ± 0.10), and 20-25 mm (0.96 ± 0.10) outside the GTV from the mean MD in mirGTV (0.93 ± 0.09). The peak values of FA and MD distributions in the GTV, peritumoral regions, and mirGTV followed similar trends (Table 2).

**Table 2:** Peak Values of the FA and MD Distributions for the GTV, Peritumoral Regions, and mirGTV *P*-values are obtained by comparing GTV and peritumoral shells to mirGTV using a two-sided paired t-test.

Structure	FA		MD (× 10^–3^ mm^2^/s)	
	Mean (range)	P	Mean (range)	P
GTV	0.089 (0.053–0.172)	0.59	1.273 (0.900–2.910)	0.02
GTV + 5 mm	0.099 (0.059–0.171)	0.97	0.940 (0.810–1.110)	0.04
GTV + 10 mm	0.112 (0.071–0.132)	0.32	0.883 (0.800–1.060)	0.04
GTV + 15 mm	0.118 (0.088–0.141)	0.13	0.851 (0.790–0.960)	0.12
GTV + 20 mm	0.125 (0.095–0.170)	0.07	0.839 (0.780–0.910)	0.32
GTV + 25 mm	0.132 (0.086–0.150)	0.03	0.820 (0.760–0.890)	0.96
mirGTV	0.100 (0.067–0.125)	—	0.830 (0.740–0.910)	—

**Figure 5:**
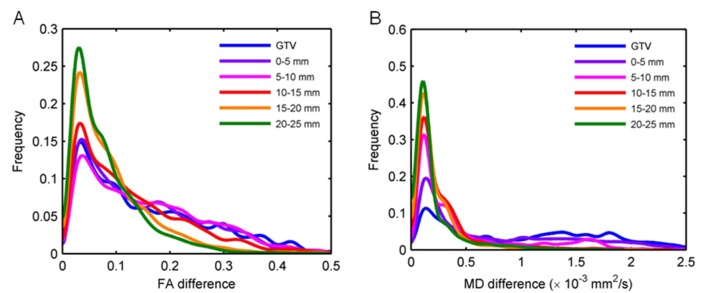
Distribution of interhemispheric differences. The distribution of absolute interhemispheric differences is shown for a glioblastoma patient for (A) fractional anisotropy (FA) and (B) mean diffusivity (MD) images.

**Figure 6:**
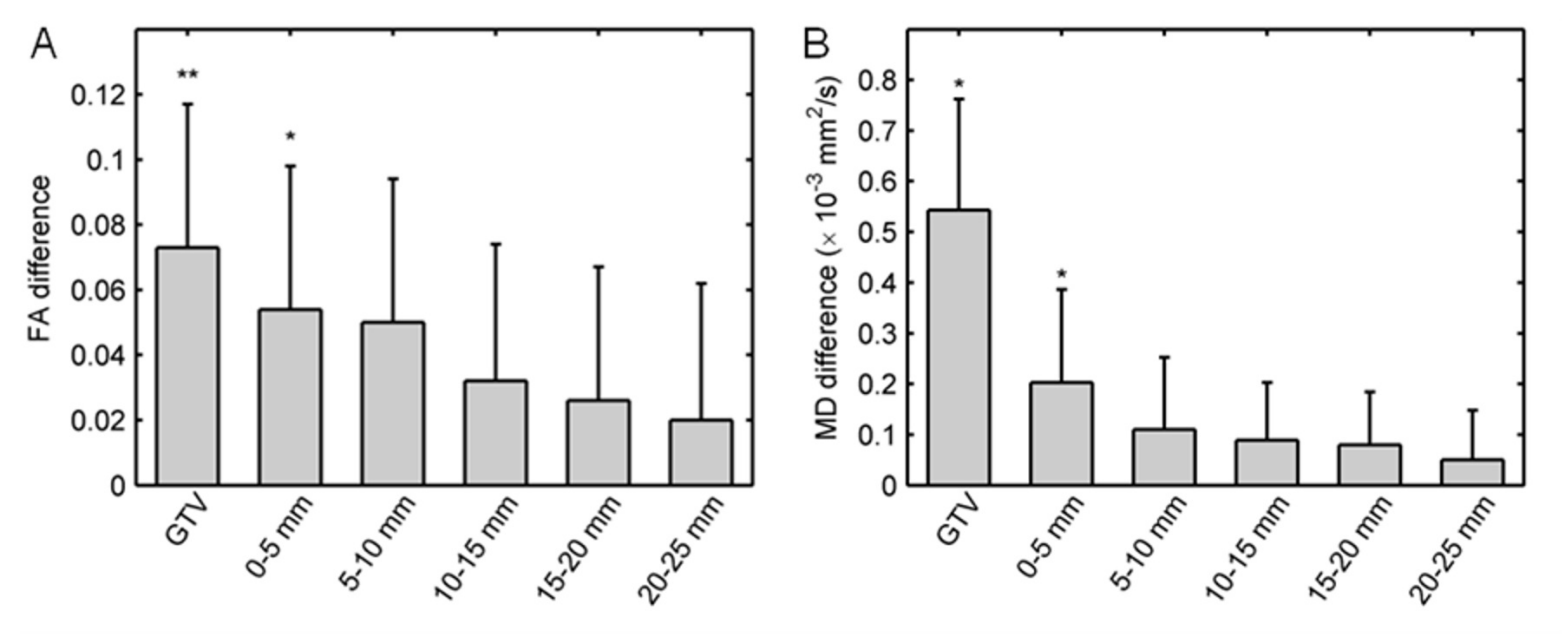
Mean interhemispheric differences. For all patients, the mean (A) fractional anisotropy (FA) and (B) mean diffusivity (MD) interhemispheric differences for the gross tumor volume (GTV) and peritumoral shells. Error bars show the standard error. Symbols: * *P *< 0.05 and ** *P *< 0.01 for two-sided paired t-tests.

Figure 5 shows the distribution of FA and MD interhemispheric differences for a sample patient. In general, the distribution of FA and MD interhemispheric differences followed the same trends as FA and MD values. Figure 6 shows the mean and standard error of FA and MD interhemispheric differences. The absolute mean FA interhemispheric differences (0.07 ± 0.04; *P* = 0.004) and mean MD interhemispheric difference (0.54 ± 0.22 × 10–3 mm^2^/s; *P* = 0.01) values were significantly larger than zero in the GTV. The mean FA and MD interhemispheric difference values were only significantly larger than zero for the 0-5 mm shell outside the GTV.

## Discussion

This study successfully implements a method to process FA and MD images of patients with high-grade gliomas. The method calculates interhemispheric difference images with peritumoral image voxel values and values obtained from anatomically homologous voxels on the contralateral side of the brain. These interhemispheric difference images provide a new way of displaying DTI images for radiation therapy planning. The area around the tumor with lower FA than contralateral normal brain was easily visualized (Figure 2A).

The MD and FA values in this study were similar to those in other studies. In this study, the FA in the GTV was 0.12 ± 0.03 and in the mirGTV was 0.20 ± 0.04. The mean MD (×10^-3^ mm^2^/s) in the GTV was 1.48 ± 0.19 and in the mirGTV was 0.93 ± 0.09. In a study of DTI of glioblastoma prior to CT-guided stereotactic biopsy, the mean values of the FA in the corpus callosum, subcortical white matter, and glioblastoma lesion were 0.70 ± 0.05, 0.32 ± 0.04, and 0.24 ± 0.05, respectively, with mean values significantly different among all three regions (*P* < 0.05) [8]. In another study, fiber density mapping and magnetic resonance spectroscopy of 48 patients with Grade II-IV glioma showed similar FA and MD values [20]. For Grade IV tumors, the FA values in the tumor, peritumoral region, and normal-appearing white matter were 0.224 ± 0.043, 0.385 ± 0.043, and 0.469 ± 0.069, and MD values (× 10^-3^ mm^2^/s) were 1.360 ± 0.164, 1.033 ± 0.107, and 0.822 ± 0.173. In a study by Khayal, et al. [21], the normalized ADC and FA for patients with glioblastoma were determined with DTI prior to, during, and following radiotherapy, with values normalized with the median ADC and FA values in the normal-appearing white matter. In the central enhancing region, the median (range) normalized ADC and FA were 0.46 (0.16 - 1.02) and 1.40 (0.29 - 2.50), respectively. Another study [22] noted similar trends in normalized FA and MD values for Grade II oligodendrogliomas, astrocytomas, and oligoastrocytomas.

Figure 4A shows an unexpected finding. The mean FA in the 20-25 mm region outside the GTV was significantly larger than the mean FA in the mirGTV. This is inconsistent with the trend of FA in the other peritumoral shells. However, on further analysis, it became clear that this increase of FA in the 20-25 mm shell occurred because this shell was more likely to include the corpus callosum. The calculation of interhemispheric FA differences eliminated this systematic anatomic variation. The mean interhemispheric FA differences in this shell were not significantly different from zero (Figure 6A), which was consistent with the trend of interhemispheric FA differences in the other peritumoral shells. This issue could also be resolved by segmenting the corpus callosum and removing the voxels that correspond to its tract from the shell volumes.

Previous studies correlated the FA and MD values with variations in tumor cell density and proliferation, but there have been conflicting reports of how they correlate. Beppu, et al. [8] and Kinoshita, et al. [9] found that FA positively correlated and MD negatively correlated with the cell density in the tumor core, while Stadlbauer, et al. [10] and Lee, et al. [11] reported that FA negatively correlated and MD positively correlated. Moreover, a study of 15 patients with high-grade gliomas found minimal anatomical overlap of the minimum ADC value, a marker of tumor cellularity, obtained from diffusion-weighted imaging (maximum *b *= 3000 s/mm^2^) and the maximum 3,4-dihydroxy-6-[^18^F]fluoro-L-phenylalanine (^18^F-FDOPA) positron emission tomography (PET) standardized uptake value ratio, a marker of tumor infiltration and proliferation [24]. Despite these inconsistent associations of FA, MD, and ADC values with pathological and imaging findings, ADC values of less than 104 × 10^–5^ mm^2^/s in non-enhancing tumor adjacent to the operative resection margin prior to temozolomide and/or chemoradiation appear to have clinical significance because they have been strongly associated with decreased overall survival (*P* < 0.001) [23].

This study has some limitations, such as the small sample size of seven patients. The true population interhemispheric variability in FA and MD may be larger than was observed in the experimental group analyzed in this cohort. There may be circumstances where this methodology would not be feasible, for example, for patients with very large surgical cavities or multifocal disease where both hemispheres have lesions or surgical cavities. The technical process of accurate fusion of postoperative imaging, particularly the nonlinear registration of the patient images with gliomas mapped to a standardized average image of healthy subjects, is required for the clinical interpretation of these images. Imperfect nonlinear image registration due to large abnormalities from the surgical cavity or naturally occurring interhemispheric differences in white matter (such as non-symmetric anatomical patterns in Wernicke's and Broca’s areas) may affect the interpretation of interhemispheric difference images. A 5-mm shell size was chosen as this is a common CTV-to-planning target volume (PTV) margin in radiotherapy planning, but FA or MD may not vary over this length scale and may result in partial volume averaging of white and gray matter within the structures.

For interhemispheric difference DTI images to be additionally useful for radiotherapy planning, it is necessary to establish by some method, such as stereotactic biopsy or comparison with three-dimensional patterns of failure, that these difference values correlate with tumor cell density or proliferation. This study also did not test the use of interhemispheric difference DTI images for radiotherapy planning in comparison to standard T1- and T2-weighted MRI, amino-acid PET imaging, spectroscopy, or exchange-mediated imaging. A quantitative analysis of target volumes contoured using DTI and other images may be performed by comparison of patterns of failure following radiotherapy, as has been performed in a previous study using ^18^F-FDOPA PET [25]. Further research will be required to determine whether these images provide information that supplements other MRI sequences or PET imaging.

## Conclusions

This study successfully implemented a method for calculation of FA and MD differences by comparison of voxel values with anatomically homologous voxels on the contralateral side of the brain. FA was significantly smaller and mean diffusivity MD was significantly larger in the GTV as compared to contralateral normal brain tissue. FA and MD values, as well as FA and MD interhemispheric differences, approached those of normal brain tissue as the distance from the GTV increased. The display of interhemispheric differences in FA and MD creates brain images that can be interpreted intuitively. Further research is warranted to determine whether interhemispheric difference images can be used to improve radiotherapy target delineation.
